# Systems-level investigation of the anxiolytic gut–brain interactions induced by paraprobiotic *Lactobacillus brevis* SBC8803 in zebrafish

**DOI:** 10.3389/fmicb.2026.1804536

**Published:** 2026-05-21

**Authors:** Azusa Kubota, Liqing Zang, Takuro Shinkai, Misa Nakai, Atsushi Tajima, Yasuhito Shimada

**Affiliations:** 1 Department of Bioinformatics and Genomics, Graduate School of Advanced Preventive Medical Sciences, Kanazawa University, Kanazawa, Japan; 2 Graduate School of Regional Innovation Studies, Mie University, Tsu, Japan; 3 Zebrafish Research Center, Mie University, Tsu, Japan; 4 Zebra-Innovate LLC, Tsu, Japan; 5 Department of Integrative Pharmacology, Graduate School of Medicine, Mie University, Tsu, Japan

**Keywords:** *Danio rerio*, host-microbe interactions, PICRUSt2, RNA-seq, teleost model

## Abstract

**Introduction:**

Anxiety disorders are among the most prevalent mental health conditions worldwide, and interest in psychobiotics—live or inactivated microorganisms that beneficially modulate the microbiota—gut–brain axis—is increasing. Heat killed *Lactobacillus brevis* SBC8803 enhances serotonin (5 hydroxytryptamine; 5 HT) signaling and ameliorates stress-related phenotypes in mammals, although the gut-brain pathways mediating these effects remain incompletely defined. Here, we investigated the anxiolytic effects and underlying molecular mechanisms of oral SBC8803 administration in adult zebrafish.

**Methods:**

Adult male AB-strain zebrafish were fed a diet containing heat killed SBC8803 for 4 weeks, and anxiety-like behavior was evaluated using the novel tank test. To explore the underlying mechanisms, we performed brain RNA sequencing and V3—V4 region of 16S rRNA amplicon sequencing of intestinal contents, followed by integrative multi omics analyses, including Gene Set Variation Analysis (GSVA) combined with DIABLO-based data integration and residual correlation analysis.

**Results:**

SBC8803-treated fish exhibited a shorter latency to enter the upper half of the tank and more frequent entries into this region, consistent with reduced anxiety-like behavior. Brain transcriptomic profiling identified differentially expressed genes and enrichment of serotonin receptor, CREB, and oxytocin signaling pathways, suggesting enhanced monoaminergic and plasticity-related signaling. Microbiome functional prediction indicated SBC8803-associated shifts in lipid and vitamin metabolism, including pathways related to riboflavin (vitamin B2) and tryptophan. GSVA combined with DIABLO-based data integration revealed coordinated changes between microbial metabolic and brain signaling pathways, consistent with a vitamin B—serotonin–anti-inflammatory axis linking gut metabolism to neural regulation. Furthermore, residual correlation analysis showed innate gut-brain coordination independent of the SBC8803 effect, such as the coupling between brain arachidonic acid and gut histidine metabolism.

**Discussion:**

These findings support the biological validity of the SBC8803 administration-associated interactions observed in the multi-omics analyses. These findings suggest that the paraprobiotic SBC8803 may exert anxiolytic-like effects in zebrafish and reshape gut—brain network states at behavioral, microbial, and transcriptomic levels, providing a potential mechanistic framework for considering heat killed SBC8803 as a candidate psychobiotic for anxiety-related conditions.

## 1 Introduction

In 2019, an estimated 301 million people were living with anxiety disorders–approximately 4% of the global population–making them the most prevalent mental health conditions worldwide (WHO, 2025). The prevalence of anxiety disorders is expected to rise further. Affected individuals typically experience persistent or excessive anxiety, worry, and fear, often accompanied by avoidance of perceived threats and panic attacks, which substantially impair daily functioning and quality of life. First-line treatments include psychotherapy (Cuijpers et al., 2024; Sun et al., 2025) and pharmacotherapy (Bandelow et al., 2017; Kong and Han, 2024), such as selective serotonin reuptake inhibitors (SSRIs), serotonin–norepinephrine reuptake inhibitors (SNRIs), tricyclic antidepressants, and benzodiazepines–are effective (Luyten et al., 2011; Mohatt et al., 2014; Østergaard, 2018). However, limitations including delayed onset of action, adverse effects, and risk of dependency underscore the need for complementary strategies.

The gut–brain axis, a bidirectional network linking the central and enteric nervous systems with the immune system and the gut microbiota, plays a fundamental role in emotional and cognitive regulation (Aburto and Cryan, 2024; He et al., 2024). Dysbiosis has been associated with anxiety-related phenotypes, potentially through serotonergic, GABAergic, and immunomodulatory pathways (Foster et al., 2017; Jiang et al., 2024). Germ-free or antibiotic-treated animals exhibit exaggerated stress responses and anxiety-like behaviors, which can be mitigated by colonization with specific commensal microorganisms (Diaz Heijtz et al., 2011; Clarke et al., 2013). Preclinical and clinical studies further suggest that modulation of the gut microbiota can reduce anxiety symptoms. Randomized trials with probiotics have reported moderate improvements, particularly with *Bifidobacterium longum* and *Lactobacillus rhamnosus* (Mosquera et al., 2024), and multiple animal and human studies indicate reductions in stress- and anxiety-related measures following probiotic intake (Gholian et al., 2024). Collectively, these findings support the concept of psychobiotics–live or inactivated microorganisms that confer mental health benefits via the gut–brain axis–as promising adjunctive interventions.

Among candidate psychobiotics, *Lactobacillus brevis* SBC8803, which was originally isolated from barley malt used in the beer brewing process (Segawa et al., 2008; Ogawa et al., 2016), is notable for its ability to enhance serotonin (5-hydroxytryptamine; 5-HT) signaling in the mammalian gut. This strain induces 5-HT release from mouse intestinal tissues and rat-derived enterochromaffin cells even in a heat-killed form (Nakaita et al., 2013), suggesting that its heat-stable structural components can stimulate the intestinal epithelium. It is generally hypothesized that such local increases in gut-derived serotonin can influence central nervous system activity via the enteric nervous system and vagal afferents, offering a potential mechanistic basis for its neuromodulatory effects. A non-randomized, double-blind, placebo-controlled crossover pilot study reported improved sleep quality following intake of heat-killed *L. brevis* SBC8803 (Nakakita et al., 2016). Additional studies have associated this strain with reduced stress-induced anxiety-like behavior and improved hippocampaus-dependent memory (Higo-Yamamoto et al., 2019; Ishikawa et al., 2019).

The present study aimed to elucidate the molecular gut–brain pathways underlying the anxiolytic effects of heat-inactivated *L. brevis* SBC8803 using zebrafish as a translational model. Adult fish received oral SBC8803 and were assessed in the novel tank diving test. To investigate underlying mechanisms, we combined brain RNA sequencing with 16S rRNA gene profiling of the gut microbiota and conducted integrative multi-omics analyses to identify host transcriptomic changes and microbial functional alterations associated with anxiolytic-like behavior.

## 2 Materials and methods

### 2.1 Zebrafish

Adult wild-type zebrafish (*Danio rerio*, AB strain, 6 months old; obtained from the Zebrafish International Resource Center, Eugene, OR, USA) were used in all experiments. The average standard length and body weight of these fish were 2.5 ± 0.23 cm and 0.28 ± 0.07 g, respectively. Fish were maintained under standard laboratory conditions, including a 14:10 h light/dark cycle, a water temperature of 28.5 °C, and a recirculating system with filtered tap water. Fish were group-housed (*n* = 10 per tank) in 2-L tanks and fed a commercial diet (Gemma Micro 300, Skretting, Stavanger, Norway) twice daily unless otherwise specified.

### 2.2 SBC8803 administration to zebrafish

To eliminate potential effects of sexual dimorphism, only adult male zebrafish were used in the feeding experiments. Five fish were housed in each 2 L tank. *Lactobacillus brevis* SBC8803 (FERM BP-10632) was obtained from the NITE Biological Resource Center (Kisarazu, Japan). The bacterial cells were cultured at 30 °C for 24 h in a broth containing 2% maltose, 1.4% yeast extract, 0.5% sodium acetate, and 0.005% MnSO4⋅5H2O. Following cultivation, the cells were harvested by centrifugation, washed three times with deionized water, and subsequently heat-killed at 105 °C for 10 min and lyophilized, as described previously (Nakaita et al., 2013). SBC8803 was incorporated into Gemma Micro 300 powder at a concentration of 2.5 × 10^10^ cells per gram of feed. The mixture was lyophilized and subsequently processed according to a previously described method to achieve a particle size of ≤700 μm (Zang et al., 2011). Each fish received 5 mg of feed per feeding (equivalent to 1.25 × 10^8^ cells), administered twice daily for 4 weeks. Because the fish consumed almost 100% of the provided diet, the daily feeding rate was approximately 3.6% of their body weight, and each fish received a daily dose of 2.5 × 10^8^ heat-killed cells, which corresponds to approximately 8.9 × 10^8^ cells per gram of body weight.

### 2.3 Novel tank diving test (NTT)

After the 4-weeks feeding period, anxiety-like behavior was assessed using a modified version of the NTT. To ensure robust evaluation of anxiety-like responses, we employed a modified protocol that consisted of three phases: acclimation, pre-test stress loading, and testing (Shinkai et al., 2025). During the acclimation phase, fish were placed in a 600-mL tank [Meito-Suien, Aichi, Japan; internal dimensions: 135 mm (width) × 95 mm (depth) × 90 mm (height)] for 5 min. Fish were then transferred to a holding tank for an additional 5 min to apply pre-test stress loading. Subsequently, fish were introduced into the test tank for behavioral assessment. The test tank had internal dimensions of 140 mm (width) × 45 mm (depth) × 120 mm (height). Behavioral recordings were captured at 30 frames per second (fps) for 5 min using a digital high-definition video camera (Panasonic HC-V495M, Osaka, Japan) positioned horizontally. Swimming behavior was analyzed using the iDTracker software (version 2.1) (Romero-Ferrero et al., 2019). Based on the refined calculation methods established in our recent study (Shinkai et al., 2025), which modified the standard behavioral indices (Stewart et al., 2012), three parameters related to anxiety-like behavior were quantified: latency to enter the top half (LTTH), frequency of entries to the top half (FE), and time spent in the top half (TSTH). In addition, the total distance traveled and average swimming speed were measured.

### 2.4 Brain RNA-sequencing

Following behavioral testing, zebrafish were euthanized by ice water immersion, and whole brains were rapidly dissected and stored in RNAlater solution (Thermo Fisher Scientific, Waltham, MA, USA). Total RNA was extracted using TRIzol reagent (Thermo Fisher Scientific) in combination with the RNeasy Mini Kit for RNA cleanup (Qiagen, Hilden, Germany). RNA integrity was assessed using a Bioanalyzer 2100 system (Agilent Technologies, Santa Clara, CA, USA), and only samples with RNA integrity number (RIN) values greater than 8.0 were used for subsequent analyses. RNA libraries were prepared according to manufacturer’s protocol and sequenced using an MGI DNBSEQ-T7 platform.

### 2.5 RNA-seq data analysis

FASTQ files were trimmed by fastp (version 0.23.4) (Chen et al., 2018). Cleaned mRNA reads were aligned to the zebrafish reference genome using STAR (version 2.7.11b) (Dobin et al., 2013). The primary genome assembly FASTA file and corresponding genomic annotation GTF file of GRCz11 (Ensembl release version 113) were used for the alignment. Transcript abundance was quantified using Salmon (version 1.10.3) (Patro et al., 2017). For transcript quantification, the reference cDNA assembly was generated from the FASTA and GTF files using gffread (version 0.12.7) (Pertea and Pertea, 2020). A comprehensive summary of sequencing and alignment statistics was compiled using MultiQC (version 1.24.dev0) (Ewels et al., 2016), integrating outputs from fastp for quality control and trimming, STAR for genomic alignment, and Salmon for transcript quantification. This integrated dataset, encompassing total read counts and alignment metrics for each sample, is provided in Supplementary Table 1. Salmon output files were imported into R and converted to raw gene counts and normalized transcripts per million (TPM) values using tximport (version 1.34.0) (Love et al., 2018). TPM values were log_2_-transformed after adding a pseudocount of 1 and used for principal component analysis (PCA). Differentially expressed gene (DEG) analysis was performed using DESeq2 (version 1.46.0) (Love et al., 2014). DEGs were identified using the following thresholds: false discovery rate (FDR) < 0.1 and absolute fold change ≥ 1.2. Annotation of DEGs was conducted using the R package biomaRt (version 2.62.1) (Smedley et al., 2009). Gene set enrichment analysis (GSEA) was performed using clusterProfiler (version 4.14.6) (Wu et al., 2021), with fold-change values derived from DESeq2 used as input. Gene sets with an FDR < 0.1 were considered significant and categorized based on the sign of their normalized enrichment score (NES). Dot plots were generated to display up to the top 10 most statistically significant pathways for both the activated (NES > 0) and suppressed (NES < 0) gene sets.

### 2.6 Ingenuity Pathway Analysis (IPA)

To perform pathway-level analysis, input gene sets for IPA were prepared from the DESeq2 results using a *p* < 0.05 and an absolute fold change ≥1.5. Distinct statistical thresholds were deliberately employed to reflect the specific objectives of each analytical step. For individual gene-level reporting, strict FDR-based criteria were used to ensure rigorous control of false positives. Conversely, for the IPA, a nominal *p*-value threshold (*p* < 0.05) was applied to provide the algorithm with a sufficiently comprehensive transcriptomic network, facilitating a more sensitive and exploratory pathway-level interpretation. Zebrafish Ensembl gene IDs were converted into their corresponding human orthologs using the BioMart database to ensure compatibility with the Ingenuity Pathway Analysis (IPA) knowledge base. The resulting DEG list was uploaded into IPA (QIAGEN, Redwood City, CA, USA), and Core Analysis was performed to identify significantly enriched canonical pathways. Pathway significance was determined using a threshold of −log(*p*-value) > 1.3, and activation states were predicted using z-scores.

### 2.7 Intestinal 16S rRNA-sequencing

Intestinal contents were collected from the zebrafish after the behavioral testing, and gut bacterial DNA was extracted using a Quick-DNA Fecal/Soil Microbe Miniprep Kit (Zymo Research, Irvine, CA, USA). The V3–V4 region of the 16S rRNA gene was amplified by PCR, and library preparation and sequencing were performed using an Illumina MiSeq platform (2 × 300 bp). Sequence data were processed using QIIME2 (version 2022.2) (Hall and Beiko, 2018), and taxonomic classification was performed using the SILVA 138 database. Functional profiles of the microbiome were inferred using Phylogenetic Investigation of Communities by Reconstruction of Unobserved Status 2 (PICRUSt2) (version 2.3.0) (Douglas et al., 2020).

### 2.8 Gene set variation analysis (GSVA)

Brain transcriptomic data and microbial PICRUSt2 output data were first converted into comparable pathway-level scores using GSVA implemented in the GSVA R package (version 2.0.7) (Hänzelmann et al., 2013). KEGG pathway gene sets for *Danio rerio* (mapped via Entrez Gene IDs) and microbial functions (mapped via KEGG Orthology identifiers) were retrieved using the KEGGREST R package (version 1.46.0). GSVA scores were calculated for each sample using the gsva function with a Gaussian kernel (kcdf = “Gaussian”), a minimum gene set size of 5, and a maximum size of 500. The resulting GSVA score matrices were used as the input for subsequent integration analyses.

### 2.9 Integrative multivariate analysis using DIABLO

Supervised multivariate analysis was performed using the DIABLO (Data Integration Analysis for Biomarker Discovery using Latent Variable Approaches for Omics Studies) framework implemented in the mixOmics R package (version 6.30.0) (Rohart et al., 2017). The GSVA score matrices for the brain (transcriptome block) and gut (microbiome_KO block) were used as predictor variables (X), with the experimental group (control vs. SBC8803) specified as the outcome variable (Y). A design matrix specifying a between-block weight of 0.1 was applied, and the number of components (ncomp) was set to 1. The optimal number of pathways to retain from each block (keepX) was determined using the tune.block.splsda function, which performed 5-fold cross-validation repeated 10 times based on the Mahalanobis distance. The search grid for keepX ranged from 10 to 50 for the transcriptome block and from 5 to 25 for the microbiome_KO block. The final block.splsda model was constructed using the optimized keepX values (20 for the transcriptome block and 25 for the microbiome_KO). Pathway loadings on component 1, representing the contribution of each selected pathway to the discrimination, were subsequently extracted.

### 2.10 Analysis of residual correlations between brain and gut pathways

Residuals adjusted for the experimental group were first calculated for each pathway from the transposed GSVA score matrices (samples × pathways) for both transcriptome and microbiome_KO blocks using linear models [lm(pathway_score ∼ group_factor)]. These residuals represent variation in pathway scores after accounting for the average effect associated with the experimental group, thereby isolating inter-individual variation independent of the main dietary intervention effect. Pairwise Spearman rank correlation coefficients and corresponding *p*-values were then computed between all brain and gut pathway residuals using the rcorr function from the Hmisc R package (version 5.2-3). The resulting *p*-value matrix was adjusted for multiple testing using the Benjamini-Hochberg procedure (p.adjust function, method = “fdr”), with an FDR < 0.05 considered statistically significant. To evaluate the robustness of these findings, a sensitivity analysis was performed in which the entire analysis pipeline, including GSVA and residual calculation, was repeated using five different pseudocount parameter values (1 × 10^–7^, 1 × 10^–6^, 1 × 10^–5^, 1 × 10^–4^, and 1 × 10^–3^) applied during the initial centered log-ratio (CLR) transformation of the KO abundance data (Gloor et al., 2017; Wang and Luan, 2024).

### 2.11 Statistical analysis

Statistical analyses for behavioral and microbial diversity data were performed using GraphPad Prism (version 10.5). For omics data, including RNA-seq and multi-omics integration, all computational analyses were conducted using R (version 4.4.3). For comparisons involving more than two groups, a one-way analysis of variance (ANOVA) followed by an appropriate *post-hoc* test was used. Data are presented as the mean ± standard deviation (SD). A *p*-value of <0.05 was considered statistically significant. Detailed statistical methodologies, specific algorithms, and thresholds (e.g., FDR) for RNA-seq, GSVA, and integrative multi-omics analyses are described in their respective subsections.

## 3 Results

### 3.1 *L. brevis* SBC8803 shows anxiolytic effects in zebrafish NTT

**FIGURE 1:**
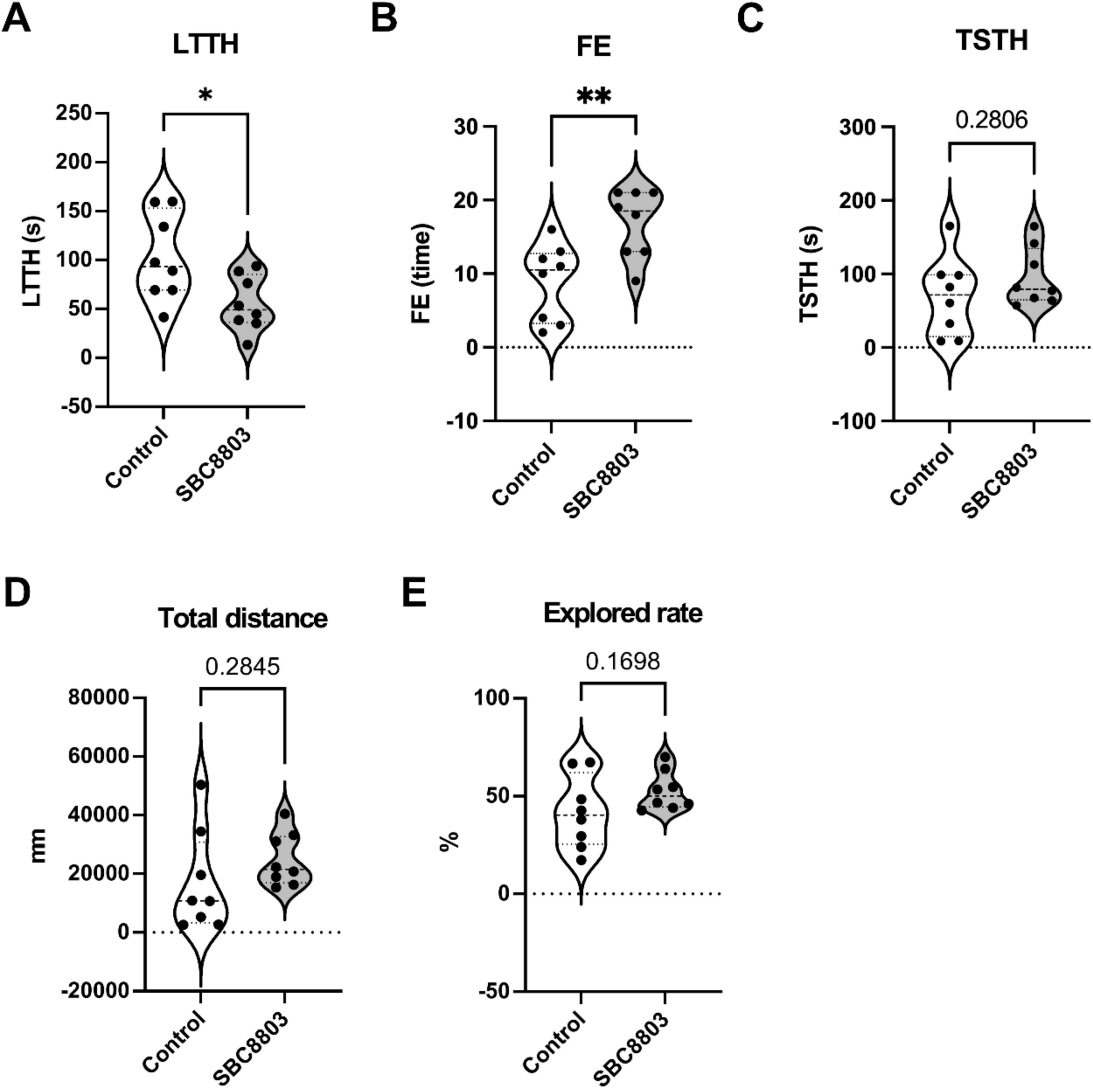
SBC8803 administration reduces anxiety-like behavior in the zebrafish novel tank test. **(A)** Latency to enter the top half of the tank (LTTH). SBC8803-treated fish exhibited a significantly shorter latency compared with control fish. **(B)** Frequency of entries into the top half of the tank (FE). SBC8803 treatment significantly increased the frequency of transitions into the upper half. **(C)** Time spent in the top half of the tank (TSTH). No significant difference was observed between groups. **(D)** Total distance traveled during the test period. No significant difference was detected between control and SBC8803-treated fish. **(E)** Exploration rate during the novel tank test. Although SBC8803-treated fish showed a tendency toward increased exploration, the difference was not statistically significant. *n* = 8 per group. Data are presented as mean ± SD. *p < 0.05, **p < 0.01; ns indicate no significant difference.

After 4 weeks of SBC8803 administration, no mortality or significant changes in body weight were observed (Supplementary Figure 1). In the NTT, SBC8803 significantly reduced latency to the top half (LTTH; *p* < 0.05; Figure 1A) and significantly increased frequency of entries to the top half (FE; *p* < 0.01; Figure 1B). In contrast, no significant difference was detected in time spent in the top half (TSTH; Figure 1C). Under control conditions, zebrafish typically exhibit stress-induced dwelling in the lower half of the tank when exposed to a novel environment. SBC8803-administrated fish showed reduced anxiety-like responses, as indicated by their shorter LTTH and increased FE. Although neither total distances traveled nor explored rate differed significantly between groups, both parameters showed a tendency to increase in SBC8803-treated fish (Figures 1D,E). These objective behavioral shifts–specifically the shorter LTTH and increased FE–indicate an anxiolytic-like response induced by SBC8803 administration, independent of basal locomotor alterations.

### 3.2 Brain transcriptome of zebrafish treated with SBC8803

To investigate transcriptomic alterations associated with the anxiolytic phenotype, whole brains were collected following SBC8803 administration and subjected to RNA sequencing. For each sample, approximately 46–117 million paired-end reads were obtained, with high-quality scores across samples (Q20: 99.1%–99.7%, Q30: 97.4%–98.6%) and an average error rate of 0.02%, ensuring sufficient depth and accuracy for transcriptome analysis. PCA of log_2_-transformed TPM values was performed to visualize global variation between groups (Supplementary Figure 2). Differential expression analysis using DESeq2 identified 128 DEGs, comprising 97 upregulated and 31 downregulated genes. These results are visualized in a volcano plot (Supplementary Figure 3) and the complete list of DEGs is provided in Supplementary Table 2. The full DESeq2 outputs is available in Supplementary Data 1.

**FIGURE 2:**
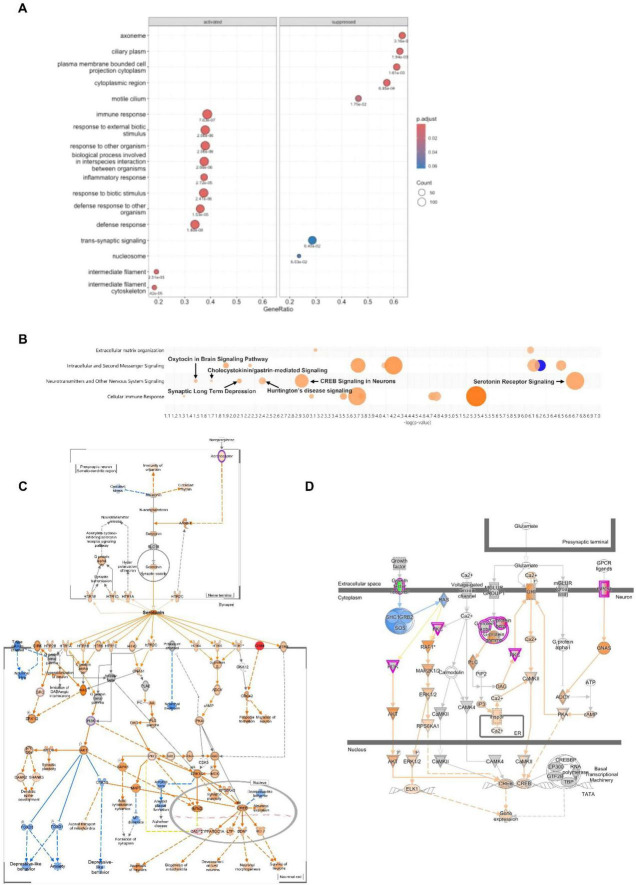
Brain transcriptomic alterations induced by SBC8803 administration. **(A)** Brain transcriptomic signatures identified by Gene Set Enrichment Analysis (GSEA). Gene Ontology (GO) enrichment analysis was performed using ranked log_2_(fold change) values derived from the DESeq2 analysis. The dot plots display the top significantly enriched GO terms (adjusted *p*-value < 0.1), categorized into activated (NES > 0) and suppressed (NES < 0) pathways. The *x*-axis represents the GeneRatio, defined as the proportion of genes contributing to the core enrichment subset relative to the total gene set size. The size of each dot corresponds to the number of enriched genes, and the color indicates the statistical significance (adjusted *p*-value: false discovery rate using Benjamini-Hochberg correction). **(B)** IPA identifies enrichment of neurotransmitter-related signaling pathways, including serotonin receptor signaling, CREB signaling in neurons, and oxytocin signaling in the brain, which may underlie the anxiolytic-like effects observed following SBC8803 treatment. All enriched pathways are shown in Supplementary Figure 4. **(C)** Predicted activation of the serotonin receptor signaling pathway in the zebrafish brain following SBC8803 administration. **(D)** Predicted activation of the canonical CREB signaling pathway in the zebrafish brain following SBC8803 administration. In panels **(C,D)**, genes with increased expression are shown in red, whereas genes with decreased expression are shown in green. Predicted activation and inhibition effects on signaling intermediates and downstream pathways are depicted by orange and blue lines, respectively, with darker hues indicating stronger predicted activation or inhibition.

To interpret the biological relevance of these transcriptomic changes, we performed GSEA using DESeq2 results. For complementary pathway-level insight, a ranked gene list based on log_2_ fold change was subjected to GO-based GSEA. In total, 102 gene sets were enriched, of which 95 were activated and 7 were suppressed based on their normalized enrichment scores (NES; Figure 2A and Supplementary Table 3).

We next applied IPA to identify signaling pathways modulated by SBC8803 administration (Supplementary Figure 4). Using thresholds of −log(*p*-value) > 1.3 and z-score > 2, six pathways within the “Neurotransmitters and Other Nervous System Signaling” category were identified as activated. This included serotonin receptor signaling, CREB signaling in neurons, Huntington’s disease signaling, synaptic long-term depression, cholecystokinin/gastrin-mediated signaling, and oxytocin signaling in the brain (Figure 2B). Ranked pathways potentially associated with mental disorders were extracted based on IPA analysis following SBC8803 treatment, using a threshold of −log(*p*-value) > 1.3 (Table 1). While IPA identified “serotonin receptor signaling” as the most strongly activated pathway [−log(*p*-value) = 6.70, z-score = 2.828; Figure 2C], the key DEGs contributing to this enrichment–such as *GNA15*, *GNB3*, *ADRB3*, and *PRKCH*–primarily encode broadly acting G protein-coupled receptor (GPCR) components and downstream second messengers. Consistent with this GPCR signal enhancement, IPA also indicated activation of the CREB signaling pathway (Figure 2D). Upstream regulators, including CaMKII, PKA, and ERK1/2, were either upregulated or predicted to be activated, converging on CREB phosphorylation and transcriptional activation.

**TABLE 1:** Ranked pathways potentially associated with mental disorders based on IPA analysis.

Ingenuity canonical pathways	−log(*p*-value)	Ratio	z-score	Molecules
Serotonin receptor signaling	6.7	0.0383	2.828	ADRB3, BLK, CASP3, CD44, F13A1, FGA, GNA15, GNB3, ITGA2B, KCNN4, LYN, PIK3R5, PLA2G4C, PRKCH, RHOG, RHOV, SNAI1, TP63
Axonal guidance signaling	3.17	0.0251	ND	GNA15, GNAT2, GNB3, ITGA2B, ITGAE, ITGB7, MYL11, NFATC2, NTRK3, PIK3R5, PLXNB2, PRKCH, VASP
Opioid signaling pathway	3.06	0.032	1.342	BLK, EGR4, GNA15, GNAT2, GNB3, LYN, OGFR, PRKCH, RGS13
nNOS signaling in neurons	3.02	0.08	ND	CAPN1, CAPN2, CAPN5, PRKCH
Neuroprotective role of THOP1 in Alzheimer’s disease	2.99	0.0462	2.236	CFD, MME, PRSS33, ST14, TMPRSS11B, TPSG1
CREB signaling in neurons	2.95	0.0227	2.714	ADGRF1, ADRB3, CCR9, CMKLR1, GNA15, GNAT2, GNB3, GPR156, GPR183, LPAR5, LTB4R, NTRK3, PIK3R5, PRKCH
Docosahexaenoic acid (DHA) signaling	2.17	0.0279	1.89	ADGRF1, CASP3, PIK3R5, PLA2G4C, PRKCH, SPTB, SYT16
Synaptic long term depression	2.09	0.0303	2.449	GNA15, GNAT2, LYN, NOS2, PLA2G4C, PRKCH
Neuroinflammation signaling pathway	2.05	0.0242	1.134	CASP3, IRF7, NCF1, NFATC2, NOS2, PIK3R5, PLA2G4C, TLR6
α-adrenergic signaling	1.78	0.036	ND	GNA15, GNAT2, GNB3, PRKCH
GABA receptor signaling	1.53	0.0301	ND	GNA15, GNAT2, GNB3, KCNN4
Oxytocin in brain signaling pathway	1.49	0.0249	2.236	GNB3, PIK3R5, PLA2G4C, PRKCH, RGS2
Reelin signaling in neurons	1.48	0.029	1	BLK, LYN, PDK2, PIK3R5
WNT/SHH axonal guidance signaling pathway	1.36	0.0265	ND	BLK, LYN, PIK3R5, TP63

ND, not determined.

### 3.3 Microbiota alteration by SBC8803 administration in zebrafish

**FIGURE 3:**
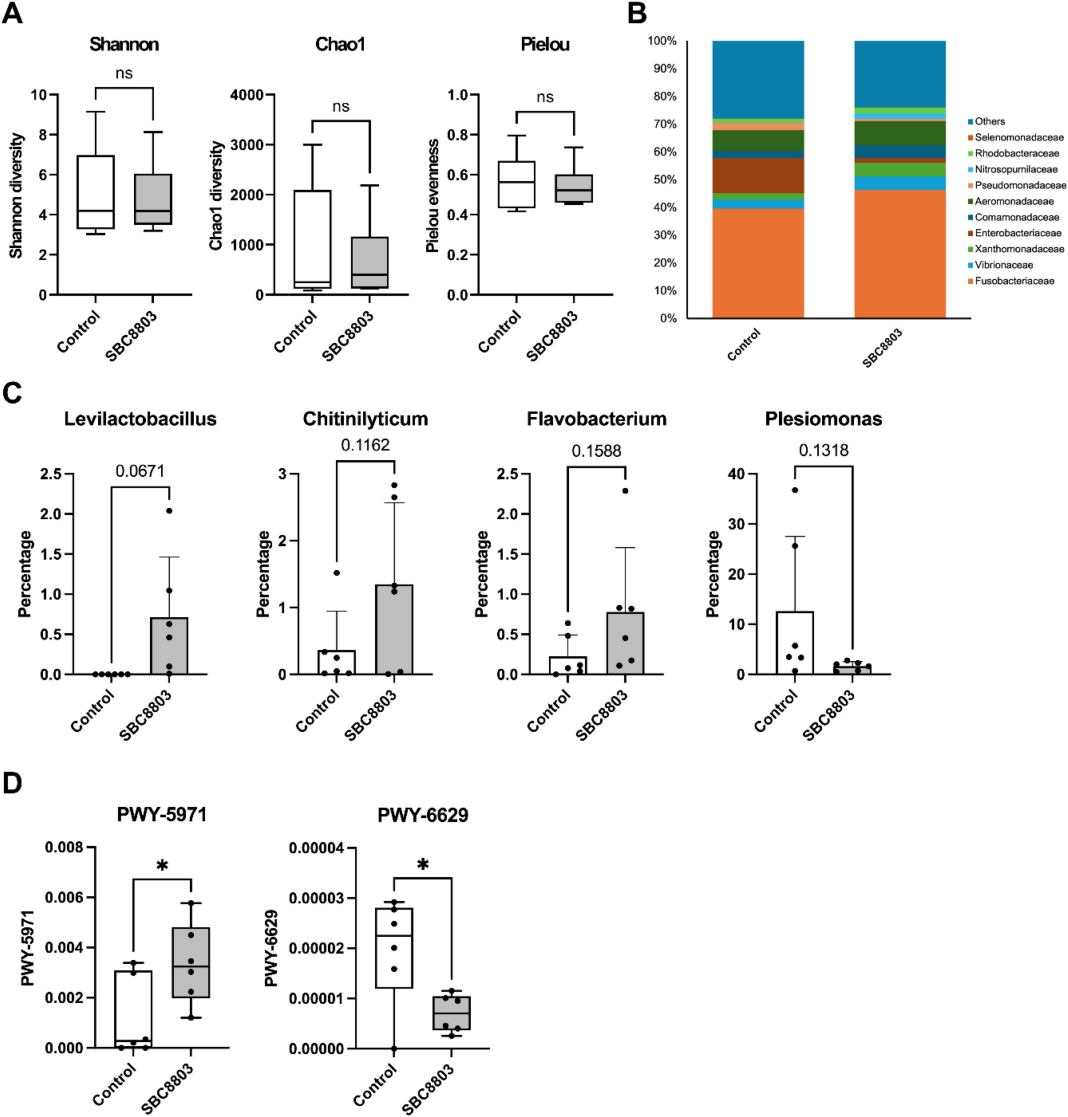
Effects of SBC8803 administration on the intestinal microbiota of zebrafish. **(A)** Alpha diversity of the gut microbiota in control and SBC8803-treated zebrafish, assessed using the Shannon diversity index, Chao1 richness, and Pielou evenness. No significant differences were observed between the two groups. **(B)** Relative abundance of dominant bacterial families in the gut microbiota of control and SBC8803-treated zebrafish. SBC8803 administration was associated with a modest increase in Fusobacteriaceae and a marked decrease in Enterobacteriaceae. **(C)** Relative abundance of bacterial genera in the gut microbiota of control and SBC8803-treated zebrafish. No genera exhibited statistically significant differences between groups (all *p* > 0.05), although *Levilactobacillus*, *Chitinilyticum*, and *Flavobacterium* tended to increase, whereas *Plesiomonas* tended to decrease following SBC8803 administration. **(D)** Functional prediction of microbial metabolic pathways inferred by PICRUSt2 and annotated using the MetaCyc database. SBC8803 administration significantly increased PWY-5971 (palmitate biosynthesis II) and significantly decreased PWY-6629 (superpathway of L-tryptophan biosynthesis). *p < 0.05.

To investigate the effects of SBC8803 on the intestinal microbiota, we profiled the gut microbiomes of control and SBC8803-treated zebrafish by amplicon sequencing of the V3–V4 region of the 16S rRNA gene. For each sample, 60,000–150,000 paired-end reads were obtained, with Q20 and Q30 scores of 98.0%–98.5% and 93.4%–94.9%, respectively. After quality filtering and processing with QIIME2, a total of 450 operational taxonomic units were identified. Despite continuous SBC8803 administration and its clear anxiolytic effect, alpha diversity was comparable between the two groups; Shannon diversity, Chao1 richness, and Pielou evenness indices showed no significant differences (Figure 3A). At the phylum level, the gut microbiota of both groups was dominated by Fusobacteria and Proteobacteria, in agreement with previous reports for adult zebrafish. Family-level profiles revealed a modest increase in Fusobacteriaceae and a marked decrease in Enterobacteriaceae in the SBC8803 group relative to controls (Figure 3B). At the genus level, no taxa displayed statistically significant differences after SBC8803 treatment (all *p* > 0.05), although *Levilactobacillus*, *Chitinilyticum*, and *Flavobacterium* tended to increase and *Plesiomonas* tended to decrease (Figure 3C). Using PICRUSt2 for functional prediction, we found that SBC8803 administration significantly increased the MetaCyc pathway PWY-5971 (palmitate biosynthesis II) and significantly decreased PWY-6629 (superpathway of L-tryptophan biosynthesis) (Figure 3D). The top 10 taxa at each taxonomic level (phylum, class, order, family, genus, and species) are summarized in Supplementary Data 2.

### 3.4 Multi-omics integration reveals coordinated gut–brain metabolic signatures

**FIGURE 4:**
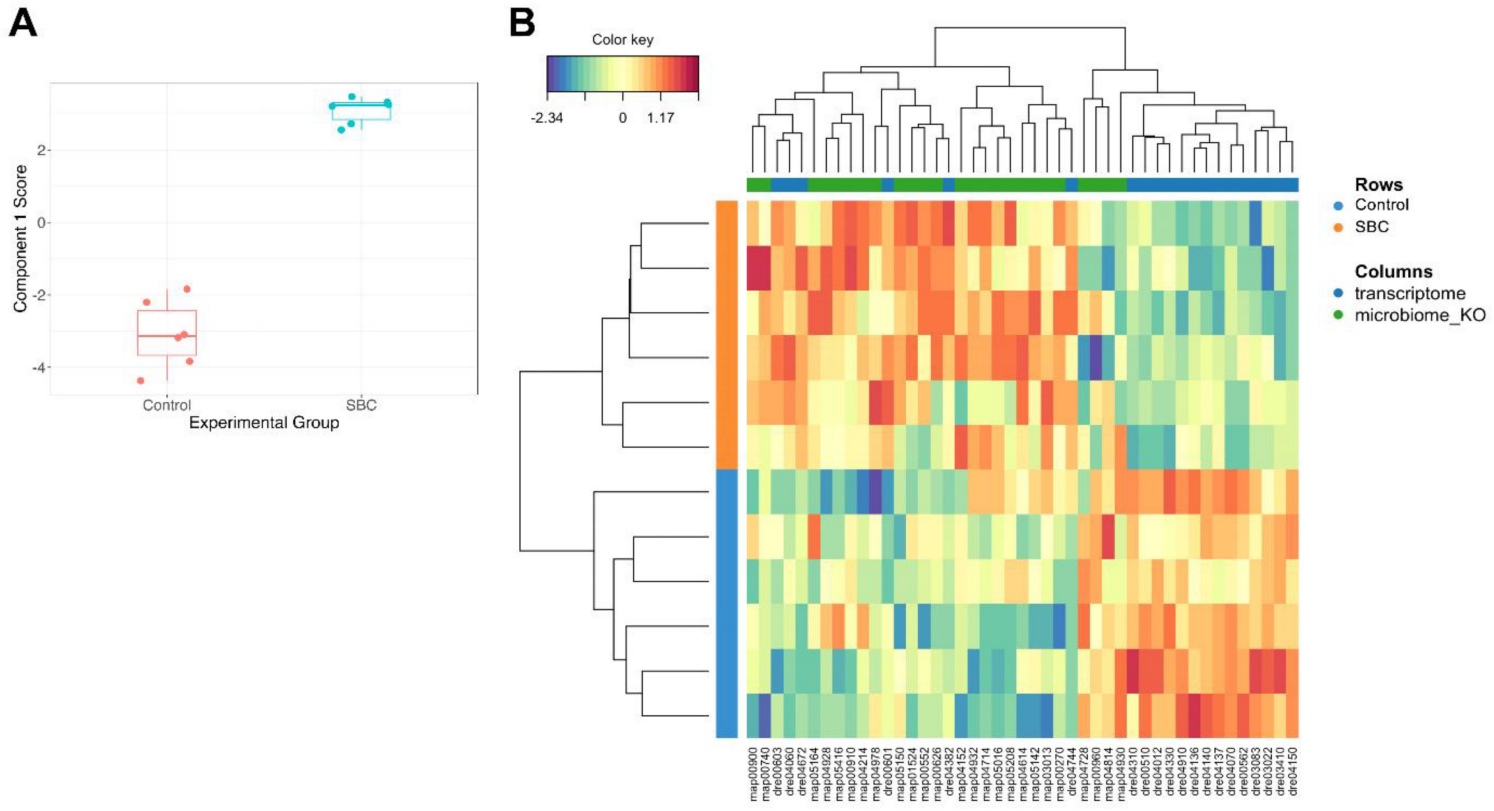
Multi-omics discrimination and molecular profiling using DIABLO. **(A)** Sample score plot for Component 1. The latent component scores, derived from the DIABLO (N-block sPLS-DA) framework, summarize the maximal covariance between the gut microbiome and brain transcriptome datasets. **(B)** Heatmap representing the cross-omics molecular profiles. The heatmap displays standardized GSVA scores for the top-ranking discriminative features selected by the model, revealing integrative classification and hierarchical clustering or the experimental groups.

Supervised integration of brain transcriptome and gut microbiome pathway scores DIABLO revealed a latent component structure strongly associated with the experimental groups (Figures 4A,B). The performance and robustness of the DIABLO model were assessed using M-fold cross-validation (5 folds, 10 repeats) during the tuning procedure. This analysis identified an optimal feature set consisting of 20 pathways from the transcriptome block and 25 pathways from the microbiome_KO block (keepX = 20 and 25, respectively), yielding a minimum mean classification error rate of 2.5% (Supplementary Figure 5).

**FIGURE 5:**
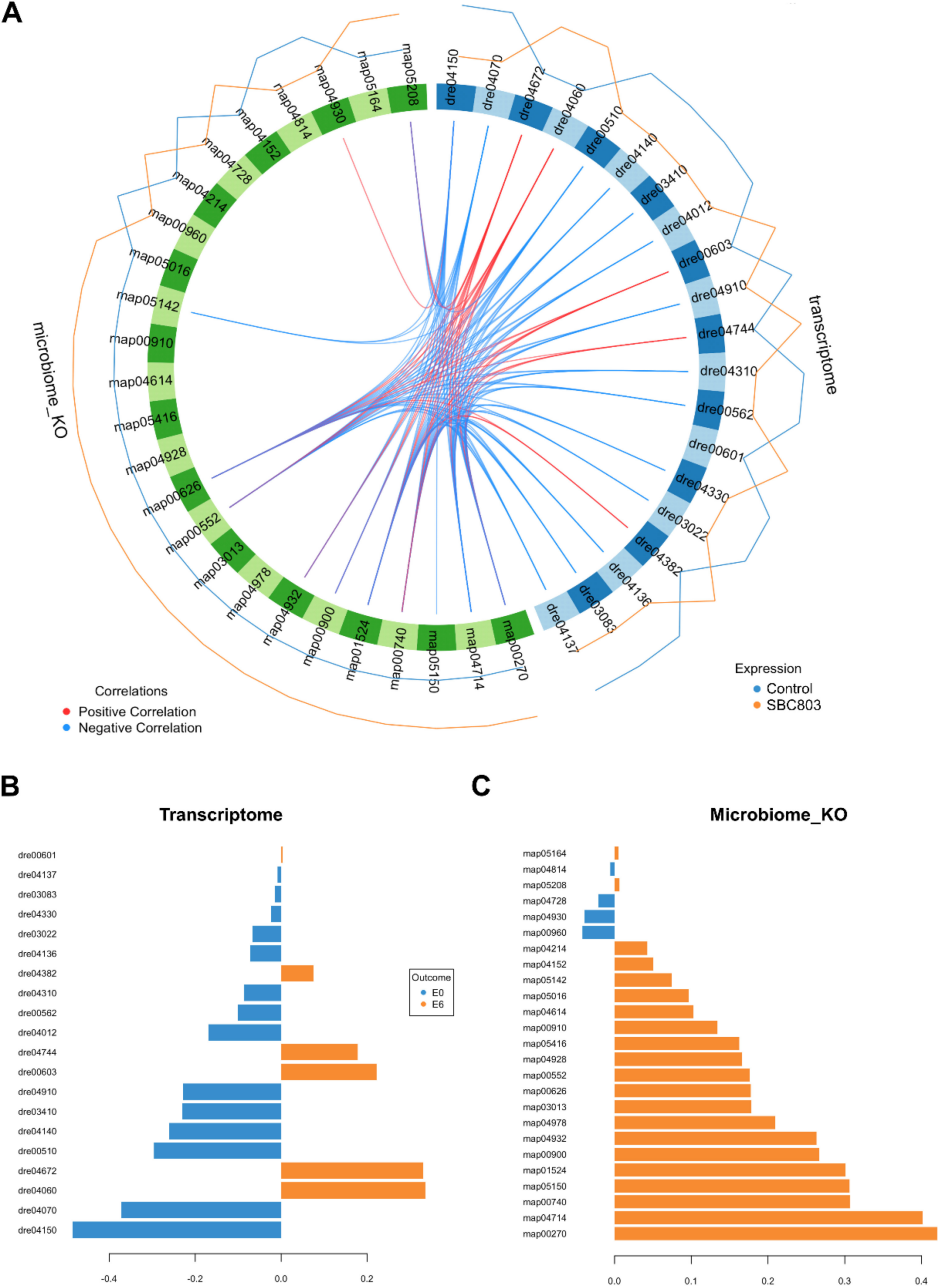
Discriminative pathway signatures and inter-modular cross-omics correlations. Identification of key microbial and brain transcriptomic features contributing to group separation and their functional associations. **(A)** Circos plot illustrating correlations between the most discriminative brain and gut pathways. Links represent strong Spearman correlations (|ρ| > 0.7), highlighting coordinated shifts between host neural pathways and microbial functional potentials identified by the DIABLO model. **(B,C)** Loading plots for the brain transcriptome **(B)** and gut microbiome **(C)**. In both omics blocks, KEGG Pathway identifiers are ranked according to their loading weights for Component 1, which represents the primary axis of separation between the control and SBC8803 groups. Colors indicate the group in which pathway activity is highest.

To visualize the cross-omics integration, correlations among the selected features from both data blocks were displayed as a circos plot (Figure 5A). Among the brain pathways, mTOR signaling pathway (dre04150; loading: −0.486) contributed most strongly to the control group, whereas cytokine-cytokine receptor interaction (dre04060; loading: +0.335) was most strongly associated with the SBC8803-treated group (Figure 5B). In the gut microbiome, the SBC8803 group was characterized by increased contributions from cysteine and methionine metabolism (map00270; loading: +0.420) and riboflavin metabolism (map00740; loading: +0.306) (Figure 5C).

### 3.5 Observation of intrinsic gut–brain coordination independent of treatment effects

**FIGURE 6:**
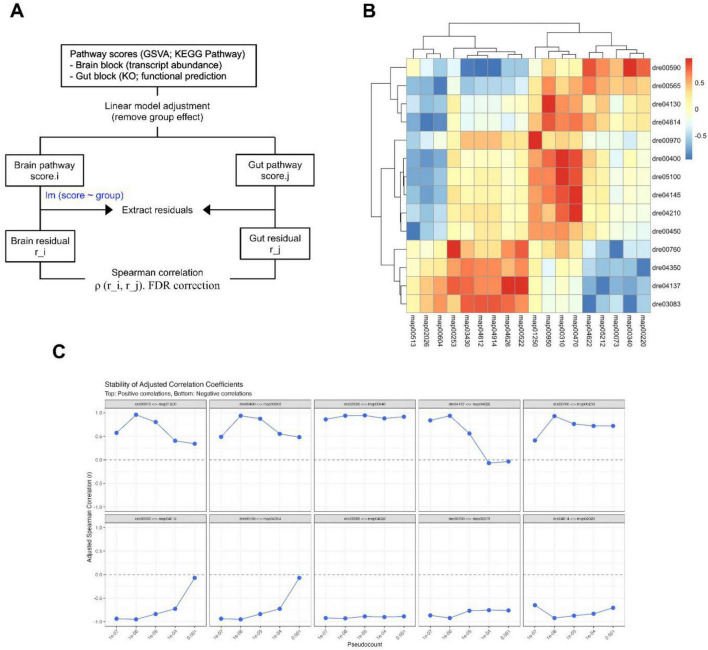
Gut–brain coordination independent of group effects shown by residual-based correlation analysis. **(A)** Conceptual overview of the residual correlation analysis workflow. To isolate gut–brain associations independent of the dietary intervention, pathway scores were adjusted using a linear model to regress out the group effect, and the resulting residuals were used for correlation analysis. **(B)** Heatmap of significant residual correlations between gut and brain pathways. Spearman’s correlation coefficients were calculated using residuals obtained after regressing out the group effect. Positive (red) and negative (blue) values indicate direct and inverse associations, respectively, independent of group-driven variations. **(C)** Sensitivity analysis of correlation stability. Plots demonstrate the robustness of adjusted Spearman’s correlation coefficients across varying pseudocount thresholds applied during the centered log-ratio (CLR) transformation.

To confirm the association between the gut–brain interactions observed by DIABLO analysis and fundamental physiological coupling, we complementarily performed Spearman correlation analysis using residuals obtained after regressing out the control/SBC8803 group effect (Figure 6A). This approach isolates inter-individual variation independent of the dietary intervention, allowing us to detect robust pathway pairs that covary across the population regardless of the treatment status. A global heatmap summarizing all associations (Supplementary Figure 6), and a total of 385 significant gut–brain pathway pairs were identified (FDR < 0.05; Supplementary Data 3).

To focus on the most robust associations, we examined the top ten positive and negative correlations ranked by effect size (Figure 6B). Among the positive associations, brain arachidonic acid metabolism (dre00590) and gut histidine metabolism (map00340) exhibited a strong correlation (ρ = + 0.94, Supplementary Figure 7, FDR = 0.0040) together with high stability across pseudocount settings (correlation range ≈ 0.08; Figure 6C). Among the negative associations, the most stable pair linked brain polycomb repressive complex–related activity (dre03083) with gut RIG-I-like receptor signaling (map04622), showing a strong inverse correlation (ρ = −0.93, FDR = 0.0053) with minimal variation in the sensitivity analysis (correlation range ≈ 0.04). Other pathway pairs, including the association between brain phenylalanine/tyrosine/tryptophan biosynthesis (dre00970) and gut secondary metabolite biosynthesis (map01250), displayed even higher coefficients (ρ = + 0.96, FDR = 0.00084) but showed lower stability across pseudocount conditions (correlation range ≈ 0.62) and were therefore interpreted with greater caution.

## 4 Discussion

This study provides an integrative view of how heat-killed *L. brevis* SBC8803 can exert psychobiotic effects through multi-layered interactions within the microbiota–gut–brain axis. Multi-omics integration revealed coordinated alterations in gut microbial function and brain transcriptional regulation, indicating a systems-level network linking microbial vitamin metabolism with host neurotransmission and immune balance. Residual correlation analysis subsequently indicated potential gut–brain association independent of SBC8803 administration, supporting the interpretation of the multi-omics analyses. Together, these findings propose a model of a metabolically mediated gut–brain crosstalk in which microbial activity related to vitamin B metabolism converges with serotonergic signaling to shape neural and immune homeostasis.

Phylogenetic Investigation of Communities by Reconstruction of Unobserved Status 2-based functional prediction illuminated the initial metabolic shifts in the gut. Palmitate biosynthesis (map00062: palmitate biosynthesis II) was upregulated, whereas the L-tryptophan biosynthesis superfamily was suppressed in the SBC8803-supplemented group. Elevated microbial palmitate biosynthesis suggests a shift toward fatty acid anabolism and membrane lipid remodeling that may influence host immune tone through altered microbial metabolite profiles. Because fatty acid synthesis requires substantial NADPH and flavin cofactors (Byrnes et al., 2018; Tolomeo et al., 2020), this pattern is consistent with the observed activation of riboflavin (vitamin B2) metabolism and redox regulation. In contrast, the predicted suppression of microbial tryptophan biosynthesis implies reduced *de novo* capacity within the gut microbiota. We hypothesize that this shift may potentially reflect a compensatory response to increased host utilization of tryptophan for the serotonin and kynurenine pathways. This complementary relationship between microbial lipid metabolism and host neurotransmitter synthesis suggests a potential coordinated redistribution of metabolic resources along the microbiota–gut–brain axis, aligning with our hypothesized “vitamin B–serotonin–anti-inflammatory” model. However, since these microbial functions were inferred using PICRUSt2 rather than directly measured via metabolomics, these links to host metabolic processes must be interpreted as proposed mechanisms requiring future experimental validation.

DIABLO multivariate analysis revealed a strong negative association between gut-derived riboflavin metabolism and brain mTOR signaling. In parallel, IPA identified activation of serotonin receptor and CREB signaling pathways in the supplemented group, suggesting enhanced synaptic plasticity-related signaling. Combined with GSEA-identified suppression of trans-synaptic signaling, these changes indicate a shift toward neural adaptation under the supplementation. This pattern is consistent with the idea that increased microbial vitamin B metabolism, particularly riboflavin, can indirectly modulate brain mTOR activity while supporting neurotransmission via serotonergic and CREB pathways (Udhayabanu et al., 2017; Ilchibaeva et al., 2022; Olfat et al., 2022). It also aligns with previous findings that vitamins B2 and B6 support redox balance and mitochondrial homeostasis, thereby mitigating stress-induced mTOR hyperactivation (Johnson et al., 2013; Ciapaite et al., 2023). Notably, while IPA highlighted upregulation of serotonin receptor signaling, this enrichment was largely supported by broadly acting GPCR-related components. Thus, these transcriptomic changes may reflect a broader modulation of general GPCR signaling networks that potentially include, but are not strictly limited to, serotonergic elements (Wong et al., 2023). Nevertheless, when considered alongside the increased expression of tcnbb (transcobalamin beta b), consistent with a role of vitamin B-dependent tryptophan hydroxylase in serotonin synthesis (Kennedy, 2016; Young et al., 2019; Tang et al., 2025). These results point to a metabolically coordinated interaction between gut-derived vitamins and host neural signaling pathways, highlighting a mechanism by which microbial metabolism shapes brain homeostasis in zebrafish.

Beyond metabolic correlations, the significant upregulation of *nfkbie*, an inhibitor of NF-κB, suggests a potential shift toward an anti-inflammatory regulatory state in the brain. While the inferred inhibition of NF-κB-related network implies that paraprobiotic treatment may engage tolerance-like mechanisms, we must emphasize that these transcriptomic observations are associative. Note that targeted proteomic or cellular assays, such as microglial morphological analysis or direct cytokine quantification, will be required to definitively confirm whether these transcriptional shifts translate into a functional reduction in neuroinflammation. This hypothesis is consistent with the GSEA results; we speculate that the enrichment of the broad “inflammatory response” and “immune response” gene sets may potentially reflect regulatory engagement by anti-inflammatory mediators rather than a purely pro-inflammatory cascade. Concomitant suppression of mTOR and MAPK signaling further reinforces this interpretation, as both pathways mediate inflammatory amplification and neuronal excitotoxicity (Switon et al., 2017; Canovas and Nebreda, 2021). Taken together, these data support a model in which microbial vitamin B metabolism indirectly enhances serotonergic activity and concurrently dampens inflammation, stabilizing neural circuits.

Previous studies provide mechanistic context for this framework. The “vitamin B–serotonin–anti-inflammatory axis” is increasingly recognized as a conduit in microbiota–gut–brain communication. Vitamins B6 and B9 (folate) act as cofactors in the enzymatic conversion of tryptophan to serotonin in the gut epithelium (Wan et al., 2022; Lu et al., 2024). Approximately 90% of total serotonin is synthesized in the gastrointestinal tract, where microbial and host metabolism jointly regulate tryptophan hydroxylase expression (González Delgado et al., 2022; Miri et al., 2023). Gut-derived serotonin not only modulates intestinal motility but also signals via vagal afferents to the brain, influencing affective and cognitive processes (Horn et al., 2022). On immune cells, activation of 5-HT2B and 5-HT4 receptors promotes an anti-inflammatory phenotype (Wan et al., 2020; Zheng and Xu, 2025); in the brain, 5-HT1A and 5-HT2A receptors activation suppresses microglial activation and oxidative stress (Krabbe et al., 2012; Glebov et al., 2015; Xing et al., 2024). Therefore, the observed upregulation of serotonergic signaling and the concurrent activation of an anti-inflammatory response (as evidenced by *nfkbie* upregulation) in our zebrafish model may underlie behavioral stabilization via suppression of neuroinflammatory tone (Whiteside et al., 1997; Demin et al., 2021). These data collectively indicate that the vitamin B–serotonin–anti-inflammatory axis not only promotes molecular homeostasis but also contributes to behavioral resilience mediated by the gut–brain interaction.

Residual correlation analysis, designed to observe the gut–brain associations independent of SBC8803’s effects, revealed two notable patterns. The first was a stable negative correlation between the brain polycomb repressive complex pathway (dre03083) and the gut RIG-I-like receptor signaling pathway (map04622). The gut RIG-I signal here represents a predicted microbial functional feature (homologs of dsRNA-recognition and RNA helicase genes) rather than activation of the host pathway. In light of the GSEA-indicated suppression of the nucleosome gene set, suggesting a shift toward less compact chromatin, reduced polycomb activity is plausible. If so, the inverse correlation implies enhanced bacterial antiviral-like functional potential in the supplemented group alongside reduced PRC activity in the brain. Given the roles of PRC1/PRC2 roles in neural development and glial maintenance in zebrafish (Raby et al., 2021; Feng and Sun, 2022; Hanot et al., 2025), this coordinated pattern may reflect a compensatory mechanism whereby microbial immune-like functional states interface with central epigenetic regulation (Liu et al., 2023). The second notable finding was a robust positive association between brain arachidonic acid metabolism (dre00590) and gut histidine metabolism (map00340). Since both arachidonic acid–derived lipids and histidine-derived metabolites modulate neuroimmune signaling (Broos et al., 2024; Dürholz et al., 2025), this covariation suggests an additional axis of coordinated gut–brain functional variation that persists beyond group-level differences. Taken together, these two findings are consistent with biological features not captured by DIABLO-based multi-omics integration, supporting the view that the paraprobiotic SBC8803 may exert additional effects beyond canonical gut–brain coupling.

Our results also suggest that paraprobiotics can elicit systemic effects comparable to those of live probiotics, in line with recent evidence. Multiple studies report that heat-killed *Lactobacillus* strains (e.g., *L. mucosae*, *L. plantarum*, *L. rhamnosus*) increase colonic IL-10, modulate serotonin receptor expression, enhance 5-HT biosynthesis, and suppress pro-inflammatory cytokines (Magryś and Pawlik, 2023; Mudaliar et al., 2024; Sun et al., 2024). These data support the view that non-viable microbial components can engage host pattern-recognition receptors and metabolic circuits, producing beneficial psychobiotic effects via immunometabolic modulation.

Several limitations should be noted. First, the associations reported here are correlational and do not establish causality; targeted metabolite supplementation or microbial genetic manipulations will be required to test causality. Second, microbial functions were predicted from 16S rRNA gene data and may not fully reflect activity; metabolomic measurements of intestinal contents and serum are needed to confirm production and circulation of vitamin-B derivatives and serotonin precursors. Third, the specific metabolic processing of the heat-killed paraprobiotic SBC8803 in the zebrafish gut remains incompletely defined. While we hypothesize that SBC8803 modulates the gut–brain axis through pathways such as short-chain fatty acid production, which directly stimulates intestinal serotonin secretion (Reigstad et al., 2015; Yano et al., 2015), the precise metabolic fate of probiotics in zebrafish requires further mapping. However, given that zebrafish models of type 2 diabetes mellitus exhibit functional microbiome profiles remarkably similar to those of humans despite taxonomic differences (Okazaki et al., 2019), it is plausible that probiotic metabolism in zebrafish largely parallels mammalian pathways. Fourth, the present study lacked a positive control, such as an established psychobiotic strain known to enhance serotonin production, due to limitations in strain availability. Future comparative studies incorporating such reference strains will be valuable for standardizing the relative efficacy of SBC8803. Finally, while zebrafish provide valuable translational insight, extrapolation warrants caution; studies in rodents or humanized models will be necessary to evaluate the generalizability of the proposed vitamin B axis and its behavioral consequences.

In summary, our data support a mechanistic framework in which gut-derived vitamins, serotonergic signaling, and anti-inflammatory pathways cooperatively sustain neural and behavioral stability. This integrated view reinforces the concept of paraprobiotics as effective psychobiotic modulators and provides a foundation for future therapeutic strategies targeting the microbiota–gut–brain axis.

## 5 Conclusion

This study demonstrates that paraprobiotic supplementation reshapes the gut–brain network through the coordinate activation of microbial vitamin B metabolism and serotonergic pathways, leading to the suppression of neuroinflammatory signaling. The integrative multi-omics approach highlights a systemic “vitamin B–serotonin–anti-inflammatory axis” linking gut metabolic function to neural gene regulation, providing a novel mechanistic basis for the psychobiotic potential of probiotics.

## Data Availability

The original contributions presented in the study are publicly available. This data can be found here: NCBI Gene Expression Omnibus (GEO), accession numbers GSE314984 (Brain RNA-seq) and GSE314985 (Intestinal 16S rRNA-seq).

## Supplementary material

Supplementary Table 1 Detailed sequencing and alignment statistics of brain RNA-seq.

Supplementary Table 2 The complete list of differentially expressed genes (DEGs).

Supplementary Table 3 List of enriched Gene Ontology (GO) terms identified by Gene Set Enrichment Analysis (GSEA).

Supplementary Data 1 The full DESeq2 outputs.

Supplementary Data 2 The top 10 taxa at each taxonomic level (phylum, class, order, family, genus, and species).

Supplementary Data 3 Significant gut–brain pathway pairs identified by residual correlation analysis.

## References

[B1] Aburto M. Cryan J. (2024). Gastrointestinal and brain barriers: Unlocking gates of communication across the microbiota-gut-brain axis. *Nat. Rev. Gastroenterol. Hepatol.* 21 222–247. 10.1038/s41575-023-00890-0 38355758

[B2] Bandelow B. Michaelis S. Wedekind D. (2017). Treatment of anxiety disorders. *Dialogues Clin. Neurosci.* 19 93–107. 10.31887/DCNS.2017.19.2/bbandelow 28867934 PMC5573566

[B3] Broos J. Y. van der Burgt R. T. M. Konings J. Rijnsburger M. Werz O. de Vries H. E. et al. (2024). Arachidonic acid-derived lipid mediators in multiple sclerosis pathogenesis: Fueling or dampening disease progression? *J. Neuroinflammation* 21:21. 10.1186/s12974-023-02981-w 38233951 PMC10792915

[B4] Byrnes J. Ganetzky R. Lightfoot R. Tzeng M. Nakamaru-Ogiso E. Seiler C. et al. (2018). Pharmacologic modeling of primary mitochondrial respiratory chain dysfunction in zebrafish. *Neurochem. Int.* 117 23–34. 10.1016/j.neuint.2017.07.008 28732770 PMC5773416

[B5] Canovas B. Nebreda A. (2021). Diversity and versatility of p38 kinase signalling in health and disease. *Nat. Rev. Mol. Cell. Biol.* 22 346–366. 10.1038/s41580-020-00322-w 33504982 PMC7838852

[B6] Chen S. Zhou Y. Chen Y. Gu J. (2018). fastp: An ultra-fast all-in-one FASTQ preprocessor. *Bioinformatics* 34 i884–i890. 10.1093/bioinformatics/bty560 30423086 PMC6129281

[B7] Ciapaite J. van Roermund C. Bosma M. Gerrits J. Houten S. IJlst L. et al. (2023). Maintenance of cellular vitamin B6 levels and mitochondrial oxidative function depend on pyridoxal 5’-phosphate homeostasis protein. *J. Biol. Chem.* 299:105047. 10.1016/j.jbc.2023.105047 37451483 PMC10463200

[B8] Clarke G. Grenham S. Scully P. Fitzgerald P. Moloney R. Shanahan F. et al. (2013). The microbiome-gut-brain axis during early life regulates the hippocampal serotonergic system in a sex-dependent manner. *Mol. Psychiatry* 18 666–673. 10.1038/mp.2012.77 22688187

[B9] Cuijpers P. Miguel C. Ciharova M. Harrer M. Basic D. Cristea I. et al. (2024). Absolute and relative outcomes of psychotherapies for eight mental disorders: A systematic review and meta-analysis. *World Psychiatry* 23 267–275. 10.1002/wps.21203 38727072 PMC11083862

[B10] Demin K. Kolesnikova T. Galstyan D. Krotova N. Ilyin N. Derzhavina K. et al. (2021). Modulation of behavioral and neurochemical responses of adult zebrafish by fluoxetine, eicosapentaenoic acid and lipopolysaccharide in the prolonged chronic unpredictable stress model. *Sci. Rep.* 11:14289. 10.1038/s41598-021-92422-6 34253753 PMC8275758

[B11] Diaz Heijtz R. Wang S. Anuar F. Qian Y. Björkholm B. Samuelsson A. et al. (2011). Normal gut microbiota modulates brain development and behavior. *Proc. Natl. Acad. Sci. U S A.* 108 3047–3052. 10.1073/pnas.1010529108 21282636 PMC3041077

[B12] Dobin A. Davis C. Schlesinger F. Drenkow J. Zaleski C. Jha S. et al. (2013). STAR: ultrafast universal RNA-seq aligner. *Bioinformatics* 29 15–21. 10.1093/bioinformatics/bts635 23104886 PMC3530905

[B13] Douglas G. Maffei V. Zaneveld J. Yurgel S. Brown J. Taylor C. et al. (2020). PICRUSt2 for prediction of metagenome functions. *Nat. Biotechnol.* 38 685–688. 10.1038/s41587-020-0548-6 32483366 PMC7365738

[B14] Dürholz K. Ehnes L. Linnerbauer M. Schmid E. Danzer H. Hinzpeter-Schmidt M. et al. (2025). Gut-specific histamine 3 receptor signaling orchestrates microglia-dependent resolution of peripheral inflammation. *J. Clin. Invest.* 135:e184697. 10.1172/JCI184697 40638239 PMC12435854

[B15] Ewels P. Magnusson M. Lundin S. Käller M. (2016). MultiQC: Summarize analysis results for multiple tools and samples in a single report. *Bioinformatics* 32 3047–3048. 10.1093/bioinformatics/btw354 27312411 PMC5039924

[B16] Feng G. Sun Y. (2022). The Polycomb group gene rnf2 is essential for central and enteric neural system development in zebrafish. *Front. Neurosci.* 16:960149. 10.3389/fnins.2022.960149 36117635 PMC9475114

[B17] Foster J. Rinaman L. Cryan J. (2017). Stress; the gut-brain axis: Regulation by the microbiome. *Neurobiol. Stress* 7 124–136. 10.1016/j.ynstr.2017.03.001 29276734 PMC5736941

[B18] Gholian M. Babaei A. Zendeboodi F. Mortazavian A. Koushki V. (2024). Ameliorating effect of psychobiotics and para-psychobiotics on stress: A review on in vivo and clinical studies and mechanism of action. *Heliyon* 10:e40338. 10.1016/j.heliyon.2024.e40338 39687128 PMC11648110

[B19] Glebov K. Löchner M. Jabs R. Lau T. Merkel O. Schloss P. et al. (2015). Serotonin stimulates secretion of exosomes from microglia cells. *Glia* 63 626–634. 10.1002/glia.22772 25451814

[B20] Gloor G. Macklaim J. Pawlowsky-Glahn V. Egozcue J. (2017). Microbiome datasets are compositional: And this is not optional. *Front. Microbiol.* 8:2224. 10.3389/fmicb.2017.02224 29187837 PMC5695134

[B21] González Delgado S. Garza-Veloz I. Trejo-Vazquez F. Martinez-Fierro M. (2022). Interplay between serotonin, immune response, and intestinal dysbiosis in inflammatory bowel disease. *Int. J. Mol. Sci.* 23:15632. 10.3390/ijms232415632 36555276 PMC9779345

[B22] Hall M. Beiko R. (2018). 16S rRNA gene analysis with QIIME2. *Methods Mol. Biol.* 1849 113–129. 10.1007/978-1-4939-8728-3_8 30298251

[B23] Hanot M. Völkel P. Le Bourhis X. Lagadec C. Angrand P. (2025). Ezh2 Loss-of-function alters zebrafish cerebellum development. *Int. J. Mol. Sci.* 26:9736. 10.3390/ijms26199736 41097006 PMC12524611

[B24] Hänzelmann S. Castelo R. Guinney J. (2013). GSVA: Gene set variation analysis for microarray and RNA-seq data. *BMC Bioinformatics* 14:7. 10.1186/1471-2105-14-7 23323831 PMC3618321

[B25] He Y. Wang K. Su N. Yuan C. Zhang N. Hu X. et al. (2024). Microbiota-gut-brain axis in health and neurological disease: Interactions between gut microbiota and the nervous system. *J. Cell. Mol. Med.* 28:e70099. 10.1111/jcmm.70099 39300699 PMC11412916

[B26] Higo-Yamamoto S. Yamamoto S. Miyazaki K. Nakakita Y. Kaneda H. Takata Y. et al. (2019). Dietary heat-killed Lactobacillus brevis SBC8803 attenuates chronic sleep disorders induced by psychophysiological stress in mice. *J. Nutr. Sci. Vitaminol.* 65 164–170. 10.3177/jnsv.65.164 31061285

[B27] Horn J. Mayer D. Chen S. Mayer E. (2022). Role of diet and its effects on the gut microbiome in the pathophysiology of mental disorders. *Transl. Psychiatry* 12:164. 10.1038/s41398-022-01922-0 35443740 PMC9021202

[B28] Ilchibaeva T. Tsybko A. Zeug A. Müller F. Guseva D. Bischoff S. et al. (2022). Serotonin receptor 5-HT2A regulates TrkB receptor function in heteroreceptor complexes. *Cells* 11:2384. 10.3390/cells11152384 35954229 PMC9368268

[B29] Ishikawa R. Fukushima H. Nakakita Y. Kado H. Kida S. (2019). Dietary heat-killed Lactobacillus brevis SBC8803 (SBL88™) improves hippocampus-dependent memory performance and adult hippocampal neurogenesis. *Neuropsychopharmacol. Rep.* 39 140–145. 10.1002/npr2.12054 30977307 PMC7292330

[B30] Jiang M. Kang L. Wang Y. Zhou B. Li H. Yan Q. et al. (2024). Mechanisms of microbiota-gut-brain axis communication in anxiety disorders. *Front. Neurosci.* 18:1501134. 10.3389/fnins.2024.1501134 39717701 PMC11663871

[B31] Johnson S. Yanos M. Kayser E. Quintana A. Sangesland M. Castanza A. et al. (2013). mTOR inhibition alleviates mitochondrial disease in a mouse model of Leigh syndrome. *Science* 342 1524–1528. 10.1126/science.1244360 24231806 PMC4055856

[B32] Kennedy D. O. (2016). B vitamins and the brain: Mechanisms, dose and efficacy–A review. *Nutrients* 8:68. 10.3390/nu8020068 26828517 PMC4772032

[B33] Kong Q. Han B. (2024). Pharmacotherapy and cognitive bias modification for the treatment of anxiety disorders. *Expert Rev. Neurother.* 24 517–525. 10.1080/14737175.2024.2334847 38557434

[B34] Krabbe G. Matyash V. Pannasch U. Mamer L. Boddeke H. Kettenmann H. (2012). Activation of serotonin receptors promotes microglial injury-induced motility but attenuates phagocytic activity. *Brain Behav. Immun.* 26 419–428. 10.1016/j.bbi.2011.12.002 22198120

[B35] Liu T. Du D. Zhao R. Xie Q. Dong Z. (2023). Gut microbes influence the development of central nervous system disorders through epigenetic inheritance. *Microbiol. Res.* 274:127440. 10.1016/j.micres.2023.127440 37343494

[B36] Love M. Huber W. Anders S. (2014). Moderated estimation of fold change and dispersion for RNA-seq data with DESeq2. *Genome Biol.* 15:550. 10.1186/s13059-014-0550-8 25516281 PMC4302049

[B37] Love M. Soneson C. Patro R. (2018). Swimming downstream: Statistical analysis of differential transcript usage following Salmon quantification. *F1000Res* 7:952. 10.12688/f1000research.15398.3 30356428 PMC6178912

[B38] Lu S. Zhao Q. Guan Y. Sun Z. Li W. Guo S. et al. (2024). The communication mechanism of the gut-brain axis and its effect on central nervous system diseases: A systematic review. *Biomed. Pharmacother.* 178:117207. 10.1016/j.biopha.2024.117207 39067168

[B39] Luyten L. Vansteenwegen D. van Kuyck K. Gabriëls L. Nuttin B. (2011). Contextual conditioning in rats as an animal model for generalized anxiety disorder. *Cogn. Affect. Behav. Neurosci.* 11 228–244. 10.3758/s13415-011-0021-6 21302154

[B40] Magryś A. Pawlik M. (2023). Postbiotic fractions of probiotics Lactobacillus plantarum 299v and Lactobacillus rhamnosus GG show immune-modulating effects. *Cells* 12:2538. 10.3390/cells12212538 37947616 PMC10648844

[B41] Miri S. Yeo J. Abubaker S. Hammami R. (2023). Neuromicrobiology, an emerging neurometabolic facet of the gut microbiome? *Front. Microbiol.* 14:1098412. 10.3389/fmicb.2023.1098412 36733917 PMC9886687

[B42] Mohatt J. Bennett S. Walkup J. (2014). Treatment of separation, generalized, and social anxiety disorders in youths. *Am. J. Psychiatry* 171 741–748. 10.1176/appi.ajp.2014.13101337 24874020

[B43] Mosquera F. Lizcano Martinez S. Liscano Y. (2024). Effectiveness of psychobiotics in the treatment of psychiatric and cognitive disorders: A systematic review of randomized clinical trials. *Nutrients* 16:1352. 10.3390/nu16091352 38732599 PMC11085935

[B44] Mudaliar S. Poojary S. Bharath Prasad A. Mazumder N. (2024). Probiotics and paraprobiotics: Effects on microbiota-gut-brain axis and their consequent potential in neuropsychiatric therapy. *Probiotics Antimicrob. Proteins* 16 1440–1464. 10.1007/s12602-024-10214-6 38294675 PMC11322360

[B45] Nakaita Y. Kaneda H. Shigyo T. (2013). Heat-Killed Lactobacillus brevis SBC8803 induces serotonin release from intestinal cells. *Food Nutr. Sci.* 4 767–771. 10.4236/fns.2013.48099

[B46] Nakakita Y. Tsuchimoto N. Takata Y. Nakamura T. (2016). Effect of dietary heat-killed Lactobacillus brevis SBC8803 (SBL88™) on sleep: A non-randomised, double blind, placebo-controlled, and crossover pilot study. *Benef. Microbes* 7 501–509. 10.3920/BM2015.0118 27013460

[B47] Ogawa M. Saiki A. Matsui Y. Tsuchimoto N. Nakakita Y. Takata Y. et al. (2016). Effects of oral intake of heat-killed Lactobacillus brevis SBC8803 (SBL88™) on dry skin conditions: A randomized, double-blind, placebo-controlled study. *Exp. Ther. Med.* 12 3863–3872. 10.3892/etm.2016.3862 28105118 PMC5228549

[B48] Okazaki F. Zang L. Nakayama H. Chen Z. Gao Z. Chiba H. et al. (2019). Microbiome alteration in Type 2 diabetes mellitus model of zebrafish. *Sci. Rep.* 9:867. 10.1038/s41598-018-37242-x 30696861 PMC6351536

[B49] Olfat N. Ashoori M. Saedisomeolia A. (2022). Riboflavin is an antioxidant: A review update. *Br. J. Nutr.* 128 1887–1895. 10.1017/S0007114521005031 35115064

[B50] Østergaard K. (2018). Treatment of selective mutism based on cognitive behavioural therapy, psychopharmacology and combination therapy - A systematic review. *Nord. J. Psychiatry* 72 240–250. 10.1080/08039488.2018.1439530 29447060

[B51] Patro R. Duggal G. Love M. Irizarry R. Kingsford C. (2017). Salmon provides fast and bias-aware quantification of transcript expression. *Nat. Methods* 14 417–419. 10.1038/nmeth.4197 28263959 PMC5600148

[B52] Pertea G. Pertea M. (2020). GFF Utilities: Gffread and GffCompare. *F1000Res* 9:304. 10.12688/f1000research.23297.2 32489650 PMC7222033

[B53] Raby L. Völkel P. Hasanpour S. Cicero J. Toillon R. Adriaenssens E. et al. (2021). Loss of polycomb repressive complex 2 function alters digestive organ homeostasis and neuronal differentiation in zebrafish. *Cells* 10:3142. 10.3390/cells10113142 34831364 PMC8620594

[B54] Reigstad C. Salmonson C. Rainey J. Szurszewski J. Linden D. Sonnenburg J. et al. (2015). Gut microbes promote colonic serotonin production through an effect of short-chain fatty acids on enterochromaffin cells. *FASEB J.* 29 1395–1403. 10.1096/fj.14-259598 25550456 PMC4396604

[B55] Rohart F. Gautier B. Singh A. Lê Cao K. (2017). mixOmics: An R package for ’omics feature selection and multiple data integration. *PLoS Comput. Biol.* 13:e1005752. 10.1371/journal.pcbi.1005752 29099853 PMC5687754

[B56] Romero-Ferrero F. Bergomi M. Hinz R. Heras F. de Polavieja G. (2019). idtracker.ai: tracking all individuals in small or large collectives of unmarked animals. *Nat. Methods* 16 179–182. 10.1038/s41592-018-0295-5 30643215

[B57] Segawa S. Nakakita Y. Takata Y. Wakita Y. Kaneko T. Kaneda H. et al. (2008). Effect of oral administration of heat-killed Lactobacillus brevis SBC8803 on total and ovalbumin-specific immunoglobulin E production through the improvement of Th1/Th2 balance. *Int. J. Food Microbiol.* 121 1–10. 10.1016/j.ijfoodmicro.2007.10.004 18055049

[B58] Shinkai T. Nakai M. Takeshita U. Morita K. Shimada Y. (2025). Refinement of the novel tank diving test: toward standardized and robust analysis of anxiety-like behavior in zebrafish. *Front. Behav. Neurosci.* 19:1624277. 10.3389/fnbeh.2025.1624277 41234536 PMC12605500

[B59] Smedley D. Haider S. Ballester B. Holland R. London D. Thorisson G. et al. (2009). BioMart–biological queries made easy. *BMC Genomics* 10:22. 10.1186/1471-2164-10-22 19144180 PMC2649164

[B60] Stewart A. Gaikwad S. Kyzar E. Green J. Roth A. Kalueff A. (2012). Modeling anxiety using adult zebrafish: A conceptual review. *Neuropharmacology* 62 135–143. 10.1016/j.neuropharm.2011.07.037 21843537 PMC3195883

[B61] Sun L. Dai X. Zhu S. Liu Z. Zhongming Z. (2025). Psychotherapies for social anxiety disorder in adults: A systematic review and Bayesian network meta-analysis. *J. Affect. Disord.* 378 301–319. 10.1016/j.jad.2025.02.092 40023260

[B62] Sun Z. Huang S. Yan X. Zhang X. Hao Y. Jiang L. et al. (2024). Living, heat-killed Limosilactobacillus mucosae and its cell-free supernatant differentially regulate colonic serotonin receptors and immune response in experimental colitis. *Nutrients* 16:468. 10.3390/nu16040468 38398793 PMC10893098

[B63] Switon K. Kotulska K. Janusz-Kaminska A. Zmorzynska J. Jaworski J. (2017). Molecular neurobiology of mTOR. *Neuroscience* 341 112–153. 10.1016/j.neuroscience.2016.11.017 27889578

[B64] Tang J. Krushelnycky L. Shaqo A. Cho C. E. (2025). A comprehensive review of nutritional influences on the serotonergic system. *Adv. Nutr.* 16:100524. 10.1016/j.advnut.2025.100524 40998119 PMC12553067

[B65] Tolomeo M. Nisco A. Leone P. Barile M. (2020). Development of novel experimental models to study flavoproteome alterations in human neuromuscular diseases: the effect of Rf therapy. *Int. J. Mol. Sci.* 21:5310. 10.3390/ijms21155310 32722651 PMC7432027

[B66] Udhayabanu T. Manole A. Rajeshwari M. Varalakshmi P. Houlden H. Ashokkumar B. (2017). Riboflavin responsive mitochondrial dysfunction in neurodegenerative diseases. *J. Clin. Med.* 6:52. 10.3390/jcm6050052 28475111 PMC5447943

[B67] Wan M. Ding L. Wang D. Han J. Gao P. (2020). Serotonin: A potent immune cell modulator in autoimmune diseases. *Front. Immunol.* 11:186. 10.3389/fimmu.2020.00186 32117308 PMC7026253

[B68] Wan Z. Zheng J. Zhu Z. Sang L. Zhu J. Luo S. et al. (2022). Intermediate role of gut microbiota in vitamin B nutrition and its influences on human health. *Front. Nutr.* 9:1031502. 10.3389/fnut.2022.1031502 36583209 PMC9792504

[B69] Wang B. Luan Y. (2024). Evaluation of normalization methods for predicting quantitative phenotypes in metagenomic data analysis. *Front. Genet.* 15:1369628. 10.3389/fgene.2024.1369628 38903761 PMC11188486

[B70] Whiteside S. Epinat J. Rice N. Israël A. (1997). I kappa B epsilon, a novel member of the I kappa B family, controls RelA and cRel NF-kappa B activity. *EMBO J.* 16 1413–1426. 10.1093/emboj/16.6.1413 9135156 PMC1169738

[B71] WHO. (2025). *Anxiety Disorders.* Available online at: https://www.who.int/news-room/fact-sheets/detail/anxiety-disorders (accessed July 28, 2025)

[B72] Wong T. Li G. Li S. Gao W. Chen G. Gan S. et al. (2023). G protein-coupled receptors in neurodegenerative diseases and psychiatric disorders. *Signal Transduct Target. Ther.* 8:177. 10.1038/s41392-023-01427-2 37137892 PMC10154768

[B73] Wu T. Hu E. Xu S. Chen M. Guo P. Dai Z. et al. (2021). clusterProfiler 4.0: A universal enrichment tool for interpreting omics data. *Innovation* 2:100141. 10.1016/j.xinn.2021.100141 34557778 PMC8454663

[B74] Xing C. Chen H. Bi W. Lei T. Hang Z. Du H. (2024). Targeting 5-HT is a potential therapeutic strategy for neurodegenerative diseases. *Int. J. Mol. Sci.* 25:13446. 39769209 10.3390/ijms252413446 PMC11679250

[B75] Yano J. Yu K. Donaldson G. Shastri G. Ann P. Ma L. et al. (2015). Indigenous bacteria from the gut microbiota regulate host serotonin biosynthesis. *Cell* 161 264–276. 10.1016/j.cell.2015.02.047 25860609 PMC4393509

[B76] Young L. Pipingas A. White D. Gauci S. Scholey A. (2019). A systematic review and meta-analysis of B vitamin supplementation on depressive symptoms, anxiety, and stress: Effects on healthy and ‘At-Risk’. *Individuals. Nutrients* 11:2232. 10.3390/nu11092232 31527485 PMC6770181

[B77] Zang L. Morikane D. Shimada Y. Tanaka T. Nishimura N. (2011). A novel protocol for the oral administration of test chemicals to adult zebrafish. *Zebrafish* 8 203–210. 10.1089/zeb.2011.0726 22181663

[B78] Zheng Y. Xu L. (2025). Bidirectional crosstalk between microglia and serotonin signaling in neuroinflammation and CNS disorders. *Front. Immunol.* 16:1646740. 10.3389/fimmu.2025.1646740 40934003 PMC12417188

